# The Story of the Insane from Year to Year

**Published:** 1896-06-27

**Authors:** 


### THE STORY OF THE INSANE FROM YEAR TO YEAR.

Ninth Series.

### THE LONDONDERRY DISTRICT ASYLUM.

It is curious to notice how almost invariably the Irish asylums are overcrowded. Here, with space for only 344, there are 425 in residence. The admissions during 1895 were 81, the recoveries 41, and the deaths 29. Calculated in the usual way the recoveries stand at 50·61 per cent., and the deaths at 6·82 per cent. Hereditary influence accounts for 20 of the year's admissions and intemperance in drink for 8; while domestic trouble is the assigned cause in 4 and love in 3. Cerebral disease was the cause of death in 7 cases and consumption in 7. The asylum seems to be in an efficient and in a happy state. The recovery rate is high; the death rate low; there was no serious accident during the year, no mechanical restraint, and no seclusion. The average cost per head per annum was £21 11s.

### ISLE OF MAN ASYLUM.

This is, we believe, the smallest public asylum in the British Isles. It contained on the date of its last report only 217 patients. There were 47 admissions, 20 recoveries, and 13 deaths. The recoveries stand at 44·6 per cent., and the deaths at 6·5. Of the admissions 10 owed their insanity to drink, in 8 the cause was unknown, and hereditary tendency does not figure at all. So far as we can trust our memory we have never before seen that cause absent from the statistical tables of our asylums. Four of the deaths were from cerebral diseases and 1 from consumption. The visitors say that "Dr. Richardson continues to manage the asylum in a most efficient and satisfactory manner." The average weekly cost per head is 8s. 7d.

